# Supplementary material for: Chiral C2-Symmetric Diimines with 4,5-Diazafluorene Units
Source: Molecules. 2019 Sep 2;24(17):3186. doi: 10.3390/molecules24173186 (PMC6749561; doi:10.3390/molecules24173186)
Supplement: Supplementary file 1 [file molecules-24-03186-s001.pdf]

Supplementary Material

Article

Chiral C_2-Symmetric Diimines with 4,5-Diazafluorene Units

Eugene S. Vasilyev [1], Sergey N. Bizyaev [1], Vladislav Yu. Komarov [2,3], Yury V. Gatilov [1] and Alexey V. Tkachev [1,3*]

[1] N. N. Vorozhtsov Novosibirsk Institute of Organic Chemistry, Siberian Branch of Russian Academy of Sciences, 9 Academician Lavrentiev Ave., 630090 Novosibirsk, Russian Federation; Vasilyev@nioch.nsc.ru (E.S.V.); bizajev@nioch.nsc.ru (S.N.B.); gatilov@nioch.nsc.ru (Yu.V.G.); atkachev@nioch.nsc.ru (A.V.T.)

[2] Nikolaev Institute of Inorganic Chemistry, Siberian Branch of Russian Academy of Sciences, Novosibirsk 630090, Russian Federation; komarov_v_y@ngs.ru

[3] Department of Natural Sciences, Novosibirsk State University, 630090 Novosibirsk, Russian Federation

* Correspondence: atkachev@nioch.nsc.ru; Fax: +7-(383) 330 9752

^{1}H and ^{13}C NMR spectra of the compounds described

4,4'-methylenebis(N-((1*R*,3*R*,8*R*,10*R*)-2,2,9,9-tetramethyl-3,4,7,8,9,10-hexahydro-1*H*-1,3:8,10-dimethanocyclopenta[1,2-*b*:5,4-*b*']diquinolin-12(2*H*)-ylidene)aniline) (2)

^{1}H NMR spectrum:

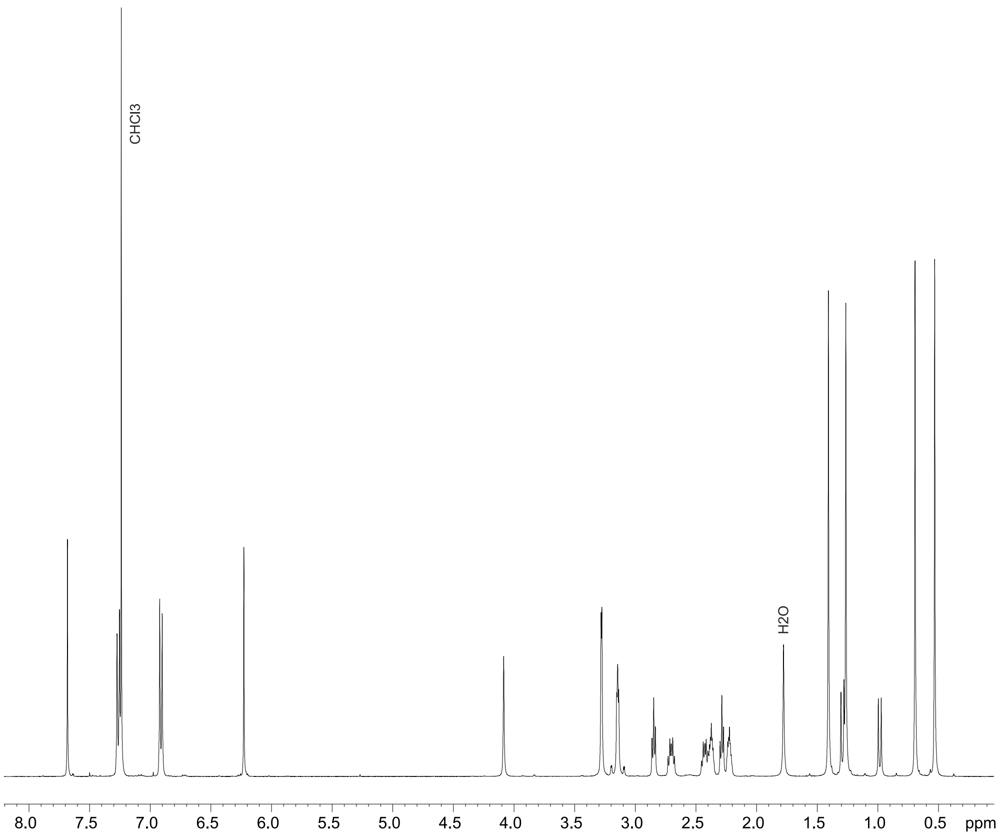

Current Data Parameters
NAME comp_2_I t s- bs- 1412-
EXPNO 1
PROCNO 1

F2 - Acquisition Parameters
Date_ 20180730
Time 17.10
INSTRUM spect
PROBHD 5 mm QNP 1H/13
PULPROG zg30
TD 32000
SOLVENT CDCl3
NS 40
DS 2
SWH 8802.817 Hz
FIDRES 0.275088 Hz
AQ 1.8176500 sec
RG 203
DW 56.800 usec
DE 6.50 usec
TE 293.6 K
D1 5.00000000 sec
TD0 11111

======== CHANNEL f1 ========
NUC1 1H
P1 13.70 usec
PL1 0 dB
PL1W 9.52005005 W
SFO1 400.1340013 MHz

F2 - Processing parameters
SI 65536
SF 400.1300183 MHz
WDW EM
SSB 0
LB 0.30 Hz
GB 0
PC 1.00

^{13}C NMR spectrum:

J-modulation,
broad-band decoupling

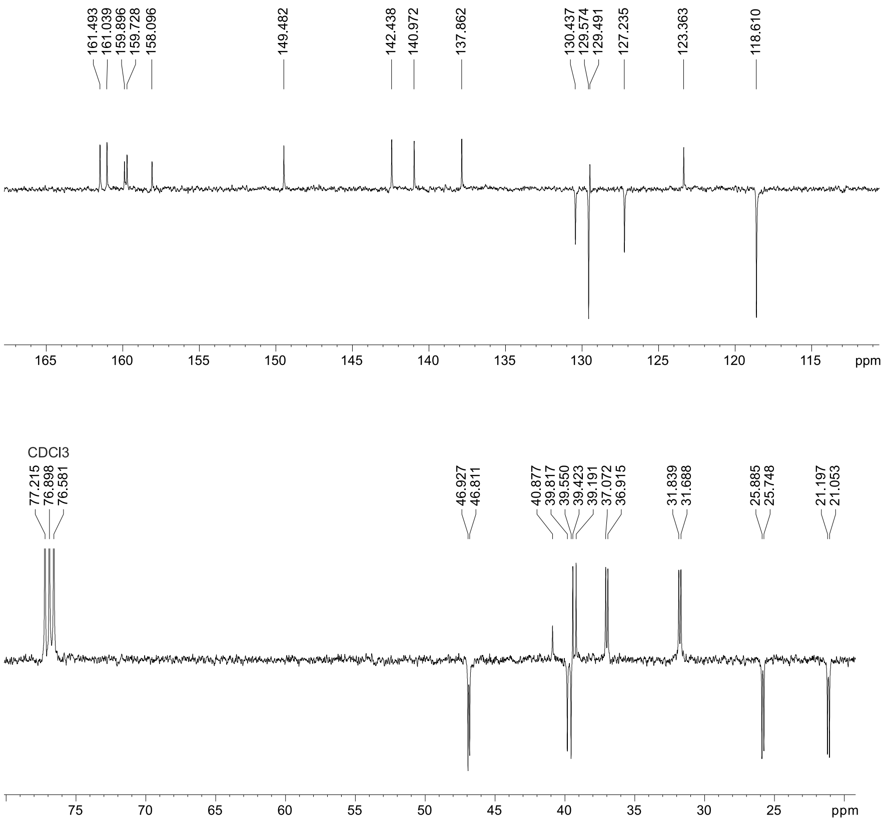

Current Data Parameters
NAME comp_2_lts-bs-1412-3
EXPNO 137
PROCNO 1

F2 - Acquisition Parameters
Date_ 20180730
Time 17.18
INSTRUM spect
PROBHD 5 mm QNP 1H/13
PULPROG jmod
TD 65536
SOLVENT CDCl3
NS 640
DS 2
SWH 24038.461 Hz
FIDRES 0.366798 Hz
AQ 1.3631988 sec
RG 203
DW 20.800 usec
DE 6.50 usec
TE 293.9 K
CNST2 145.0000000
CNST11 1.0000000
D1 5.00000000 sec
D20 0.00689655 sec
TD0 11111

======== CHANNEL f1 ========
NUC1 13C
P1 9.60 usec
P2 19.20 usec
PL1 0 dB
PL1W 36.42235184 W
SFO1 100.6243395 MHz

======== CHANNEL f2 ========
CPDPRG2 waltz16
NUC2 1H
PCPD2 86.00 usec
PL2 0 dB
PL12 16.00 dB
PL2W 9.52005005 W
PL12W 0.23913284 W
SFO2 400.1316005 MHz

F2 - Processing parameters
SI 131072
SF 100.6127842 MHz
WDW EM
SSB 0
LB 3.00 Hz
GB 0
PC 1.40

3,3',5,5'-tetramethyl-N4,N4'-bis((1*R*,3*R*,8*R*,10*R*)-2,2,9,9-tetramethyl-3,4,7,8,9,10-hexahydro-1*H*-1,3:8,10-dimethanocyclopenta[1,2-*b*:5,4-*b*']diquinolin-12(2*H*)-ylidene)-[1,1'-biphenyl]-4,4'-diamine (3)

^{1}H NMR spectrum:

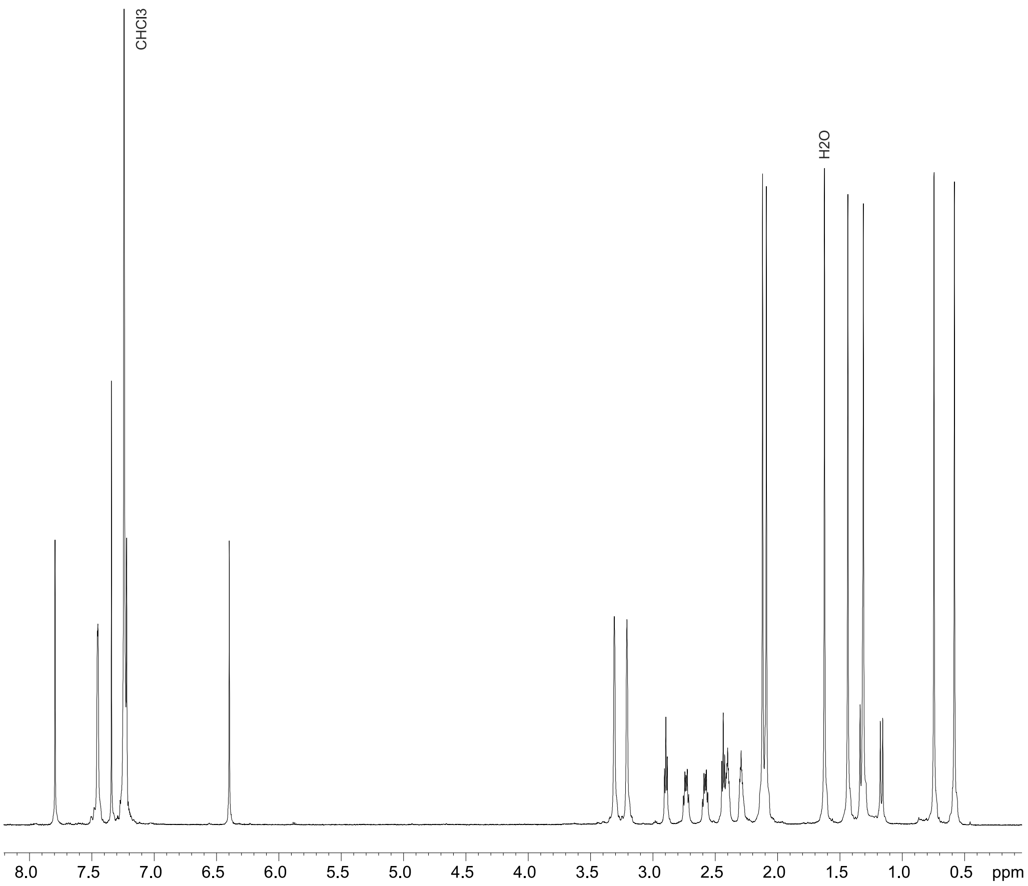

Current Data Parameters
NAME comp_3_l t s- bs- 1419-
EXPNO 1
PROCNO 1

F2 - Acquisition Parameters
Date_ 20180913
Time 12.30
INSTRUM spect
PROBHD 5 mm Multinucl
PULPROG zg30
TD 65536
SOLVENT CDCl3
NS 40
DS 2
SWH 10964.912 Hz
FIDRES 0.167311 Hz
AQ 2.9884915 sec
RG 406.4
DW 45.600 usec
DE 6.00 usec
TE 300.0 K
D1 5.00000000 sec
TD0 11111

======== CHANNEL f1 ========
NUC1 1H
P1 13.75 usec
PL1 -3.00 dB
SFO1 500.1347512 MHz

F2 - Processing parameters
SI 65536
SF 500.1300235 MHz
WDW EM
SSB 0
LB 0.30 Hz
GB 0
PC 1.00

^{13}C NMR spectrum:

J-modulation,
broad-band decoupling

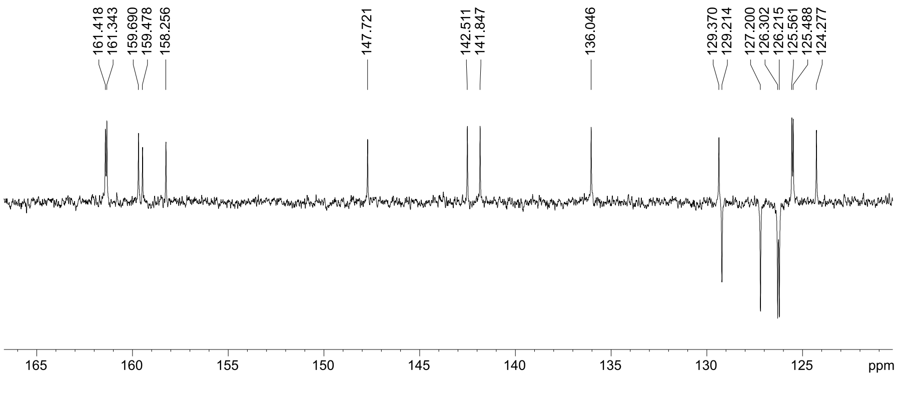

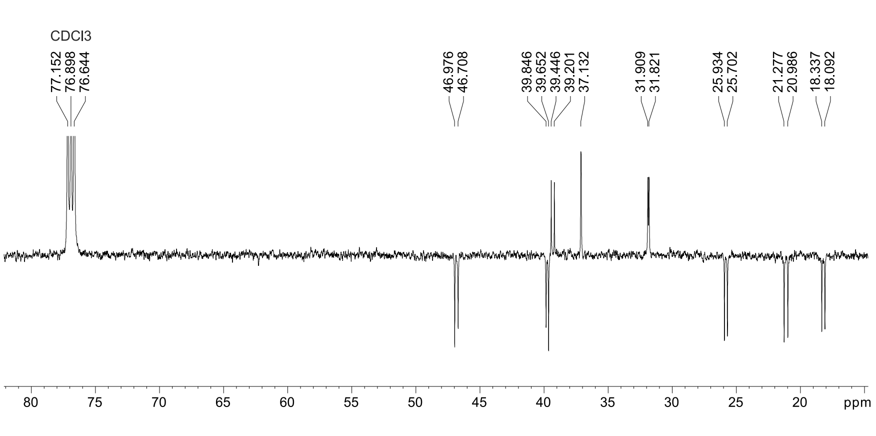

Current Data Parameters
NAME comp_3_lts-bs-1419-4
EXPNO 137
PROCNO 1

F2 - Acquisition Parameters
Date_ 20180913
Time 12.38
INSTRUM spect
PROBHD 5 mm Multinucl
PULPROG jmod
TD 65536
SOLVENT DMSO
NS 3872
DS 4
SWH 30303.031 Hz
FIDRES 0.462388 Hz
AQ 1.0813940 sec
RG 8192
DW 16.500 usec
DE 6.00 usec
TE 300.0 K
CNST2 145.0000000
CNST11 1.0000000
D1 4.00000000 sec
d20 0.00689655 sec
DELTA 0.00001184 sec
TD0 11111

======== CHANNEL f1 ========
NUC1 13C
P1 9.30 usec
p2 18.60 usec
PL1 5.00 dB
SFO1 125.7720754 MHz

======== CHANNEL f2 ========
CPDPRG2 waltz16
NUC2 1H
PCPD2 76.50 usec
PL2 -3.00 dB
PL12 13.00 dB
SFO2 500.1320005 MHz

F2 - Processing parameters
SI 131072
SF 125.7578067 MHz
WDW EM
SSB 0
LB 3.00 Hz
GB 0
PC 1.00

N1,N4-bis((1*R*,3*R*,8*R*,10*R*)-2,2,9,9-tetramethyl-3,4,7,8,9,10-hexahydro-1*H*-1,3:8,10-dimethanocyclopenta[1,2-*b*:5,4-*b*']diquinolin-12(2*H*)-ylidene)benzene-1,4-diamine (4)

^{1}H NMR spectrum:

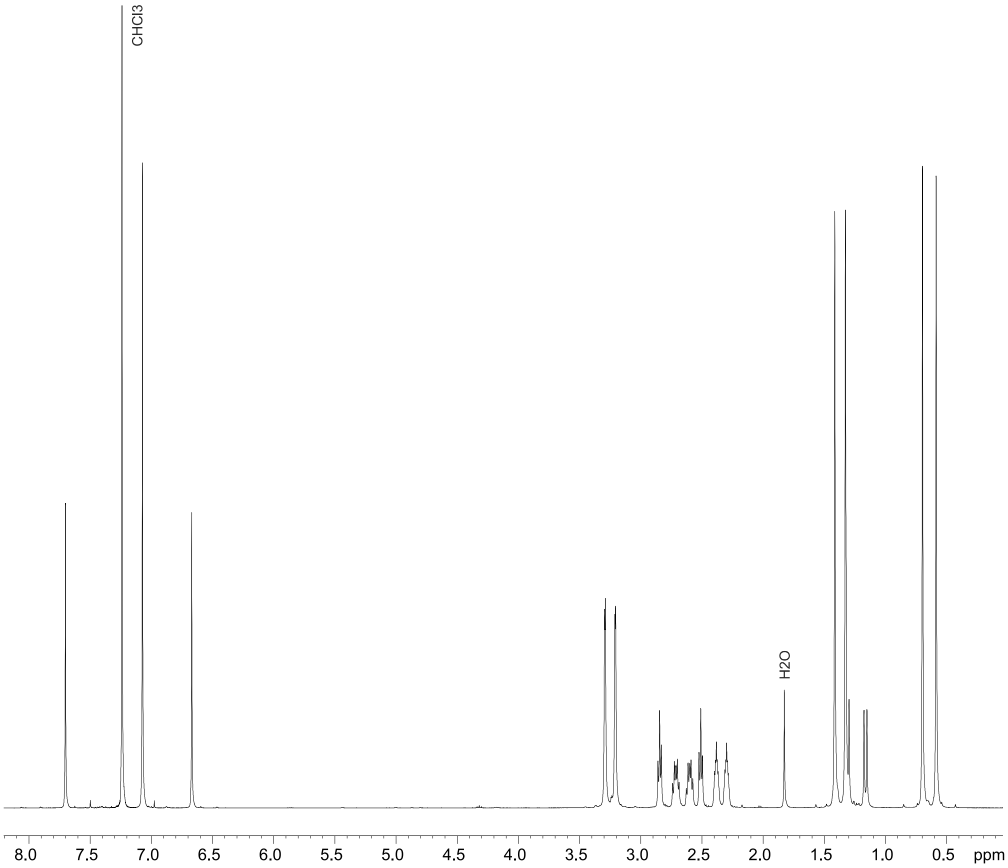

Current Data Parameters
NAME comp_4_l t s- bs- 1422-
EXPNO 1
PROCNO 1

F2 - Acquisition Parameters
Date_ 20181004
Time 18.25
INSTRUM spect
PROBHD 5 mm QNP 1H/13
PULPROG zg30
TD 32000
SOLVENT CDCl3
NS 64
DS 2
SWH 8802.817 Hz
FIDRES 0.275088 Hz
AQ 1.8176500 sec
RG 203
DW 56.800 usec
DE 6.50 usec
TE 292.3 K
D1 5.00000000 sec
TD0 11111

======== CHANNEL f1 ========
NUC1 1H
P1 13.70 usec
PL1 0 dB
PL1W 9.52005005 W
SFO1 400.1340013 MHz

F2 - Processing parameters
SI 65536
SF 400.1300185 MHz
WDW EM
SSB 0
LB 0.30 Hz
GB 0
PC 1.40

^{13}C NMR spectrum:

J-modulation,
broad-band decoupling

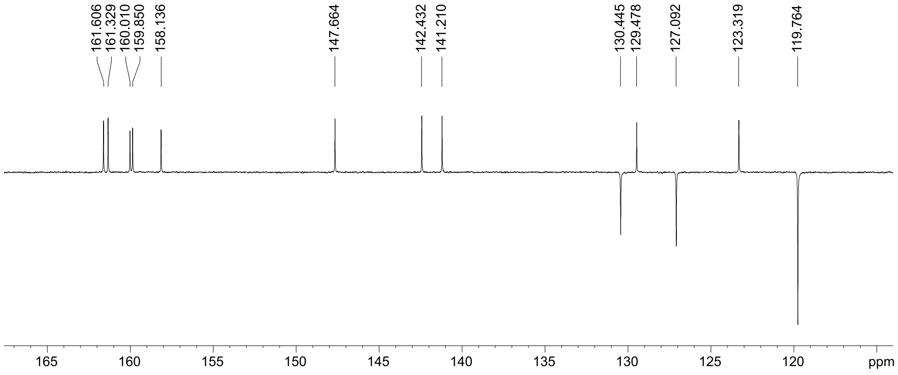

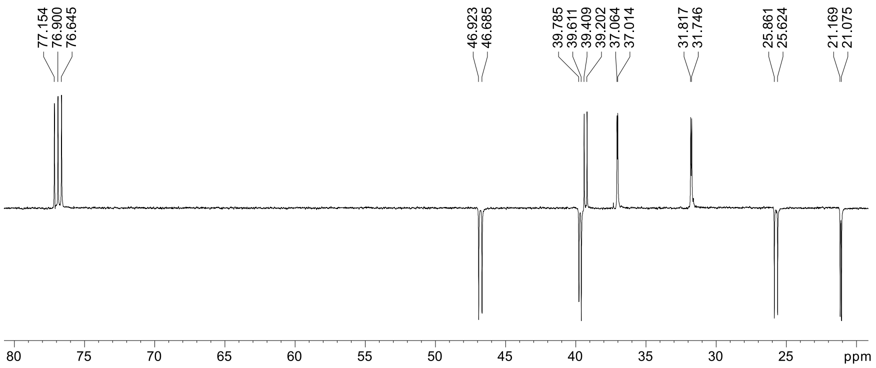

Current Data Parameters
NAME comp_4_lts-bs-1422-1_C
EXPNO 137
PROCNO 1

F2 - Acquisition Parameters
Date_ 20181003
Time 11.42
INSTRUM spect
PROBHD 5 mm Multinucl
PULPROG jmod
TD 65536
SOLVENT CDCl3
NS 1264
DS 4
SWH 30303.031 Hz
FIDRES 0.462388 Hz
AQ 1.0813940 sec
RG 3251
DW 16.500 usec
DE 6.00 usec
TE 300.0 K
CNST2 145.0000000
CNST11 1.0000000
D1 4.00000000 sec
d20 0.00689655 sec
DELTA 0.00001184 sec
TD0 11111

======== CHANNEL f1 ========
NUC1 13C
P1 9.30 usec
p2 18.60 usec
PL1 5.00 dB
SFO1 125.7720754 MHz

======== CHANNEL f2 ========
CPDPRG2 waltz16
NUC2 1H
PCPD2 76.50 usec
PL2 -3.00 dB
PL12 13.00 dB
SFO2 500.1320005 MHz

F2 - Processing parameters
SI 131072
SF 125.7578116 MHz
WDW EM
SSB 0
LB 2.00 Hz
GB 0
PC 1.00

N1,N3-bis((1*R*,3*R*,8*R*,10*R*)-2,2,9,9-tetramethyl-3,4,7,8,9,10-hexahydro-1*H*-1,3:8,10-dimethanocyclopenta[1,2-*b*:5,4-*b*']diquinolin-12(2*H*)-ylidene)benzene-1,3-diamine (5)

^{1}H NMR spectrum:

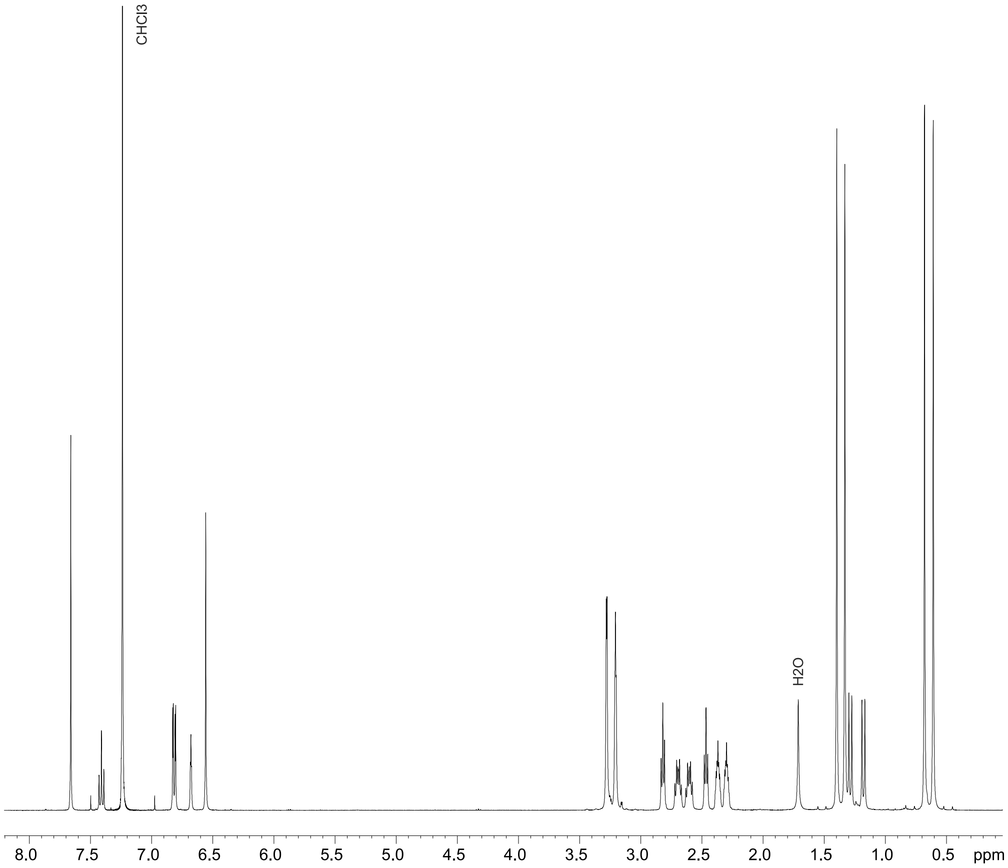

Current Data Parameters
NAME comp_5_l t s- bs- 1428-
EXPNO 1
PROCNO 1

F2 - Acquisition Parameters
Date_ 20181010
Time 12.20
INSTRUM spect
PROBHD 5 mm QNP 1H/13
PULPROG zg30
TD 32768
SOLVENT CDCl3
NS 96
DS 2
SWH 8802.817 Hz
FIDRES 0.268641 Hz
AQ 1.8612725 sec
RG 40.3
DW 56.800 usec
DE 6.50 usec
TE 292.7 K
D1 5.00000000 sec
TD0 256

======== CHANNEL f1 ========
NUC1 1H
P1 13.70 usec
PL1 0 dB
PL1W 9.52005005 W
SFO1 400.1340013 MHz

F2 - Processing parameters
SI 65536
SF 400.1300182 MHz
WDW EM
SSB 0
LB 0.18 Hz
GB 0
PC 1.40

^{13}C NMR spectrum:

J-modulation,
broad-band decoupling

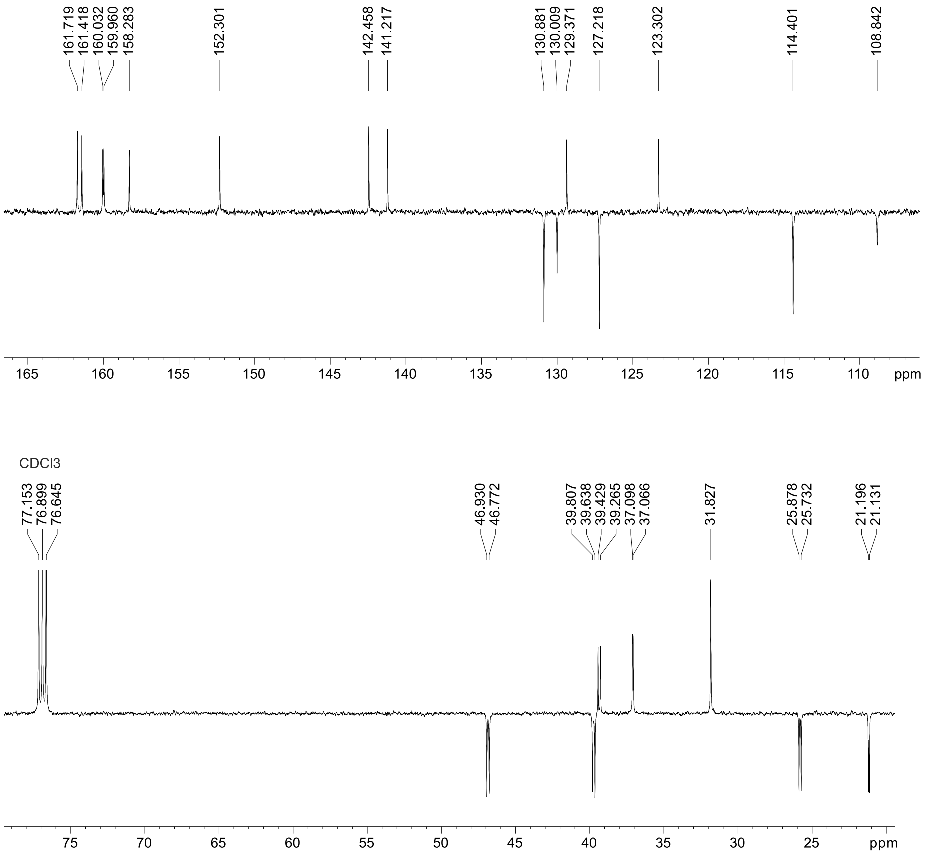

Current Data Parameters
NAME comp_5_lts-bs-1428-1_C
EXPNO 137
PROCNO 1

F2 - Acquisition Parameters
Date_ 20181010
Time 14.04
INSTRUM spect
PROBHD 5 mm Multinucl
PULPROG jmod
TD 65536
SOLVENT DMSO
NS 1968
DS 4
SWH 30303.031 Hz
FIDRES 0.462388 Hz
AQ 1.0813940 sec
RG 5160.6
DW 16.500 usec
DE 6.00 usec
TE 300.0 K
CNST2 145.0000000
CNST11 1.0000000
D1 4.00000000 sec
d20 0.00689655 sec
DELTA 0.00001184 sec
TD0 11111

======== CHANNEL f1 ========
NUC1 13C
P1 9.30 usec
p2 18.60 usec
PL1 5.00 dB
SFO1 125.7720754 MHz

======== CHANNEL f2 ========
CPDPRG2 waltz16
NUC2 1H
PCPD2 76.50 usec
PL2 -3.00 dB
PL12 13.00 dB
SFO2 500.1320005 MHz

F2 - Processing parameters
SI 131072
SF 125.7578071 MHz
WDW EM
SSB 0
LB 3.00 Hz
GB 0
PC 1.00

N1,N2-bis((1*R*,3*R*,8*R*,10*R*)-2,2,9,9-tetramethyl-3,4,7,8,9,10-hexahydro-1*H*-1,3:8,10-dimethanocyclopenta[1,2-*b*:5,4-*b*']diquinolin-12(2*H*)-ylidene)benzene-1,2-diamine (6)

^{1}H NMR spectrum:

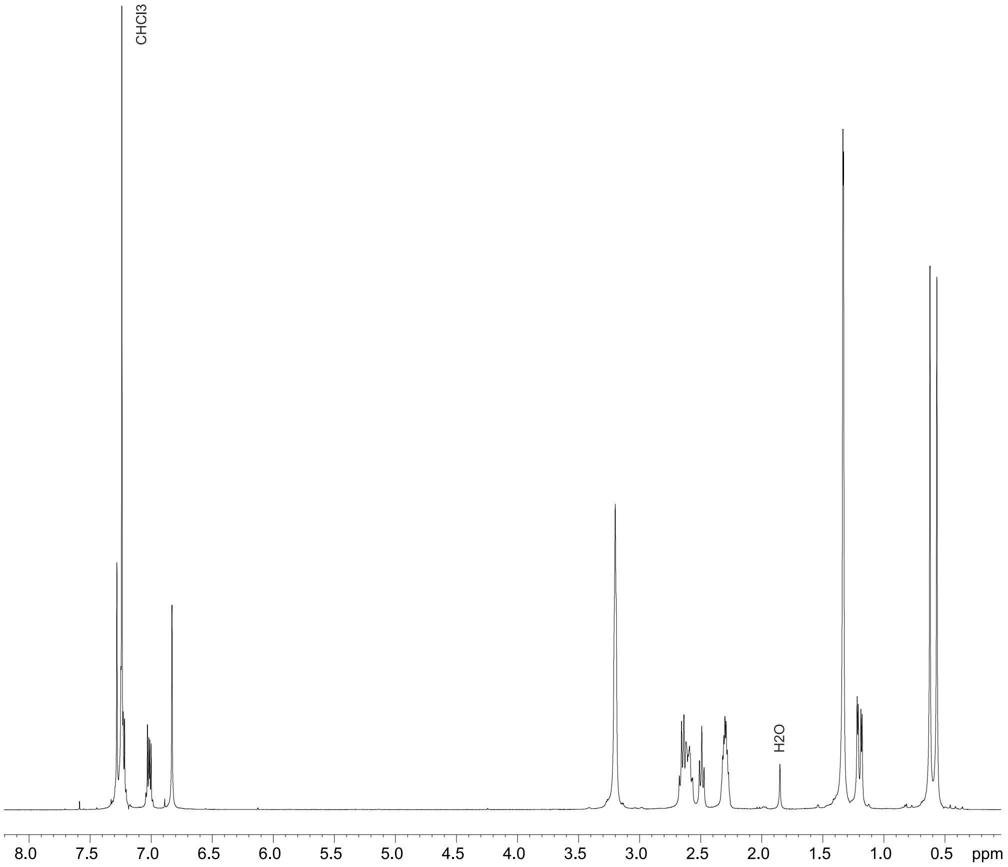

Current Data Parameters
NAME comp_6_l t s- bs- 1435-
EXPNO 1
PROCNO 1

F2 - Acquisition Parameters
Date_ 20181121
Time 12.50
INSTRUM av300
PROBHD 5 mm QNP 1H/13
PULPROG zg
TD 32768
SOLVENT CDCl3
NS 64
DS 2
SWH 4401.409 Hz
FIDRES 0.134320 Hz
AQ 3.7224948 sec
RG 90.5
DW 113.600 usec
DE 6.00 usec
TE 296.5 K
D1 10.00000000 sec
TD0 1024

======== CHANNEL f1 ========
NUC1 1H
P1 14.90 usec
PL1 0 dB
PL1W 9.79635715 W
SFO1 300.1318733 MHz

F2 - Processing parameters
SI 65536
SF 300.1300127 MHz
WDW GM
SSB 0
LB -0.50 Hz
GB 0.1
PC 1.00

^{13}C NMR spectrum:

J-modulation,
broad-band decoupling

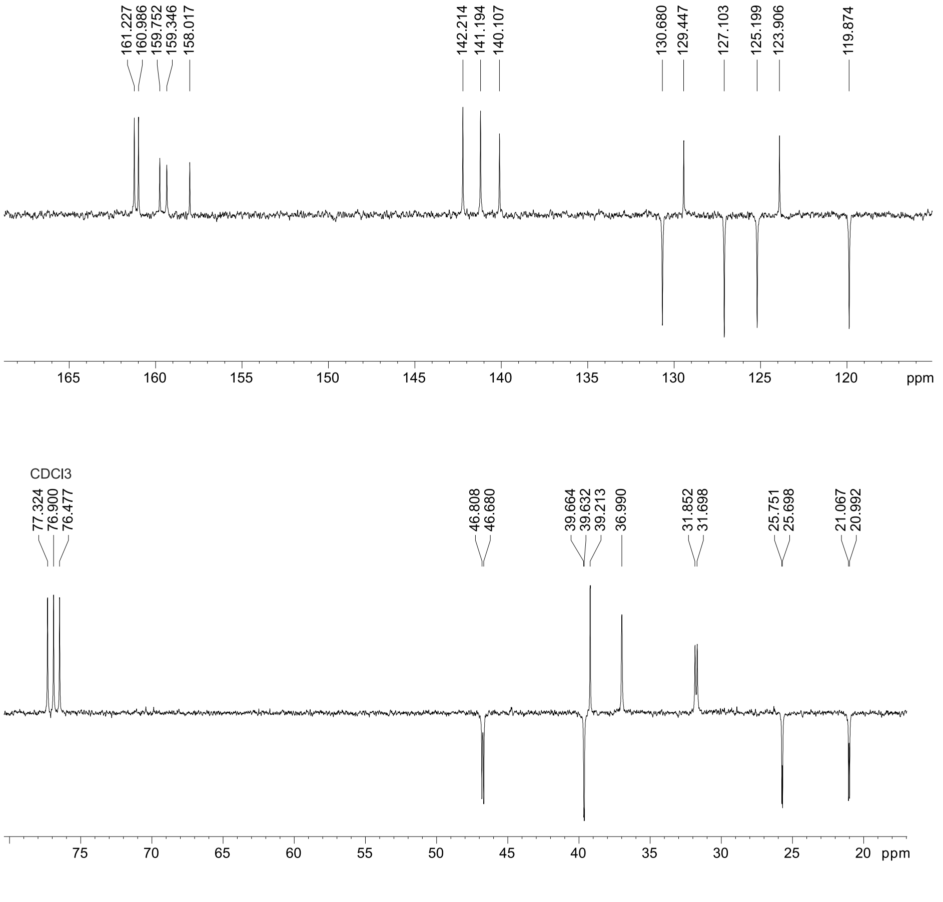

Current Data Parameters
NAME comp_6_lts-bs-1435-1_C_75
EXPNO 137
PROCNO 1

F2 - Acquisition Parameters
Date_ 20181116
Time 16.35
INSTRUM av300
PROBHD 5 mm QNP 1H/13
PULPROG jmod
TD 32768
SOLVENT CDCl3
NS 304
DS 4
SWH 18115.941 Hz
FIDRES 0.552855 Hz
AQ 0.9044468 sec
RG 32768
DW 27.600 usec
DE 6.00 usec
TE 297.2 K
CNST2 145.0000000
CNST11 1.0000000
D1 5.00000000 sec
D20 0.00689655 sec
TD0 11111

======== CHANNEL f1 ========
NUC1 13C
P1 11.20 usec
P2 22.40 usec
PL1 1.00 dB
PL1W 27.73414612 W
SFO1 75.4760505 MHz

======== CHANNEL f2 ========
CPDPRG2 waltz16
NUC2 1H
PCPD2 74.50 usec
PL2 0 dB
PL12 14.00 dB
PL2W 9.79635715 W
PL12W 0.38999999 W
SFO2 300.1312005 MHz

F2 - Processing parameters
SI 65536
SF 75.4677624 MHz
WDW EM
SSB 0
LB 2.00 Hz
GB 0
PC 1.40

N1,N5-bis((1*R*,3*R*,8*R*,10*R*)-2,2,9,9-tetramethyl-3,4,7,8,9,10-hexahydro-1*H*-1,3:8,10-dimethanocyclopenta[1,2-*b*:5,4-*b*']diquinolin-12(2*H*)-ylidene)naphthalene-1,5-diamine (7)

^{1}H NMR spectrum:

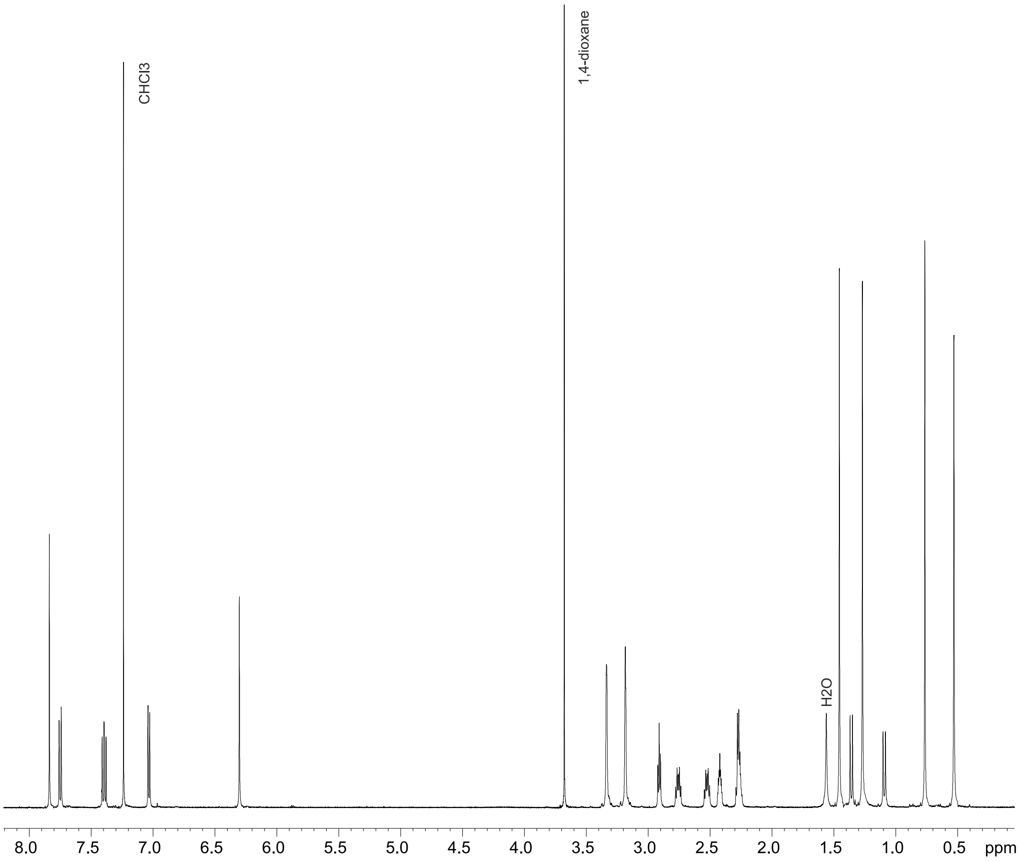

Current Data Parameters
NAME comp_7_l t s- bs- 1509-
EXPNO 1
PROCNO 1

F2 - Acquisition Parameters
Date_ 20190506
Time 15.47
INSTRUM spect
PROBHD 5 mm Multinucl
PULPROG zg30
TD 65536
SOLVENT CDCl3
NS 32
DS 2
SWH 10964.912 Hz
FIDRES 0.167311 Hz
AQ 2.9884915 sec
RG 512
DW 45.600 usec
DE 6.00 usec
TE 303.0 K
D1 5.00000000 sec
TD0 11111

======== CHANNEL f1 ========
NUC1 1H
P1 13.75 usec
PL1 -3.00 dB
SFO1 500.1347512 MHz

F2 - Processing parameters
SI 131072
SF 500.1300234 MHz
WDW no
SSB 0
LB 0 Hz
GB 0
PC 1.00

^{13}C NMR spectrum:

J-modulation,
broad-band decoupling

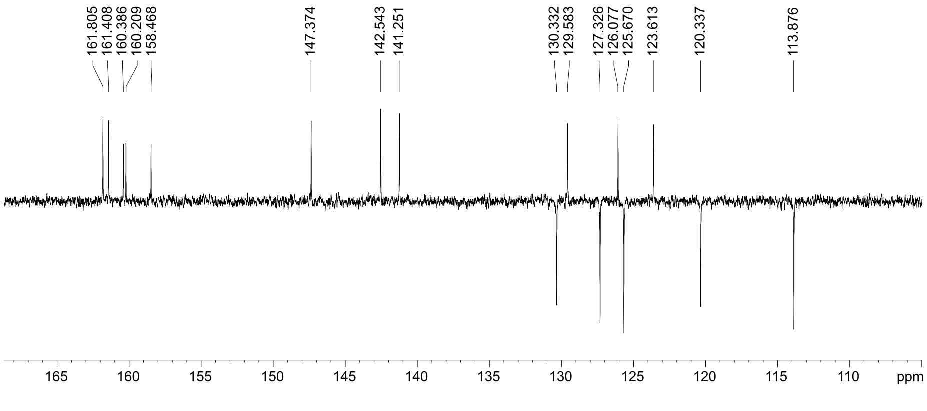

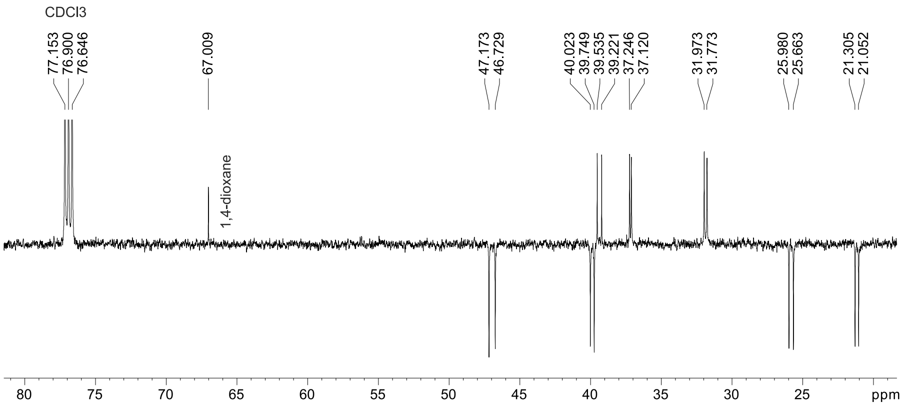

Current Data Parameters
NAME comp_7_lts-bs-1509-19
EXPNO 137
PROCNO 1

F2 - Acquisition Parameters
Date_ 20190506
Time 15.53
INSTRUM spect
PROBHD 5 mm Multinucl
PULPROG jmod
TD 65536
SOLVENT CDCl3
NS 1984
DS 4
SWH 30303.031 Hz
FIDRES 0.462388 Hz
AQ 1.0813940 sec
RG 5160.6
DW 16.500 usec
DE 6.00 usec
TE 303.0 K
CNST2 145.0000000
CNST11 1.0000000
D1 4.00000000 sec
d20 0.00689655 sec
DELTA 0.00001184 sec
TD0 11111

======== CHANNEL f1 ========
NUC1 13C
P1 9.30 usec
p2 18.60 usec
PL1 5.00 dB
SFO1 125.7720754 MHz

======== CHANNEL f2 ========
CPDPRG2 waltz16
NUC2 1H
PCPD2 76.50 usec
PL2 -3.00 dB
PL12 13.00 dB
SFO2 500.1320005 MHz

F2 - Processing parameters
SI 131072
SF 125.7577999 MHz
WDW EM
SSB 0
LB 2.00 Hz
GB 0
PC 1.00

1,2-bis((1*R*,3*R*,8*R*,10*R*)-2,2,9,9-tetramethyl-3,4,7,8,9,10-hexahydro-1*H*-1,3:8,10-dimethanocyclopenta[1,2-*b*:5,4-*b*']diquinolin-12(2*H*)-ylidene)hydrazine (8)

^{1}H NMR spectrum:

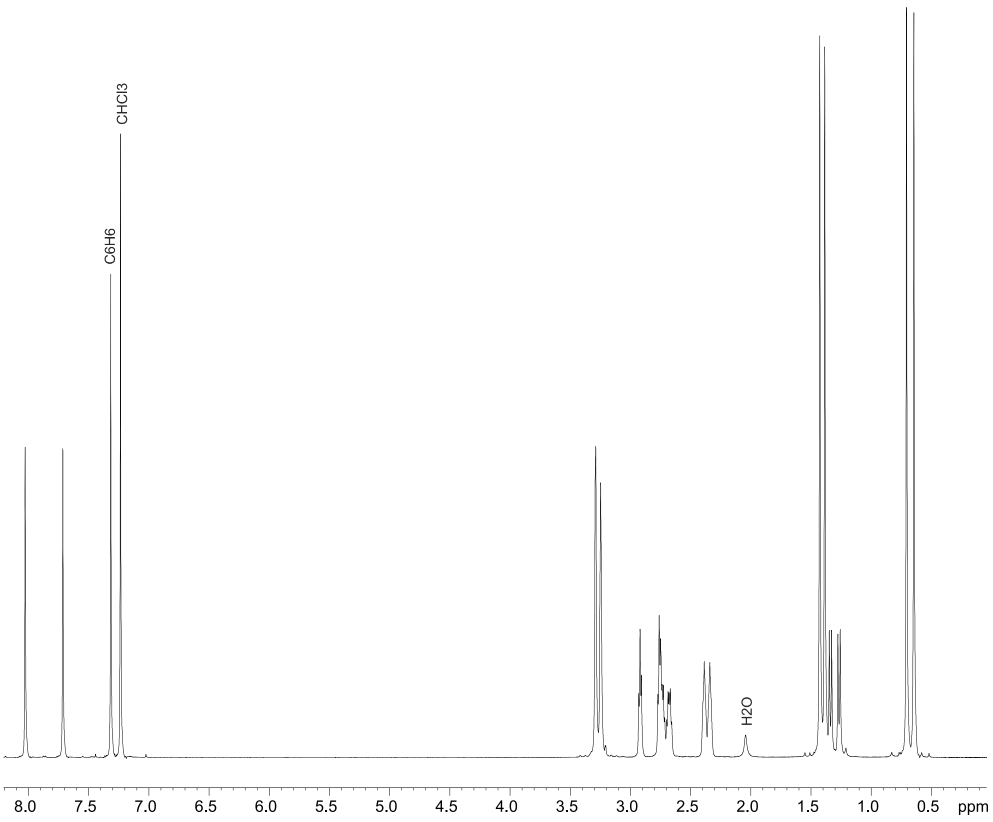

Current Data Parameters
NAME comp_8_lts-bs-1430-
EXPNO 1
PROCNO 1

F2 - Acquisition Parameters
Date_ 20181015
Time 14.34
INSTRUM spect
PROBHD 5 mm Multinucl
PULPROG zg30
TD 65536
SOLVENT CDCl3
NS 16
DS 2
SWH 10964.912 Hz
FIDRES 0.167311 Hz
AQ 2.9884915 sec
RG 101.6
DW 45.600 usec
DE 6.00 usec
TE 300.0 K
D1 5.00000000 sec
TD0 11111

======== CHANNEL f1 ========
NUC1 1H
P1 13.75 usec
PL1 -3.00 dB
SFO1 500.1347512 MHz

F2 - Processing parameters
SI 65536
SF 500.1300265 MHz
WDW EM
SSB 0
LB 0.30 Hz
GB 0
PC 1.00

^{13}C NMR spectrum:

J-modulation,
broad-band decoupling

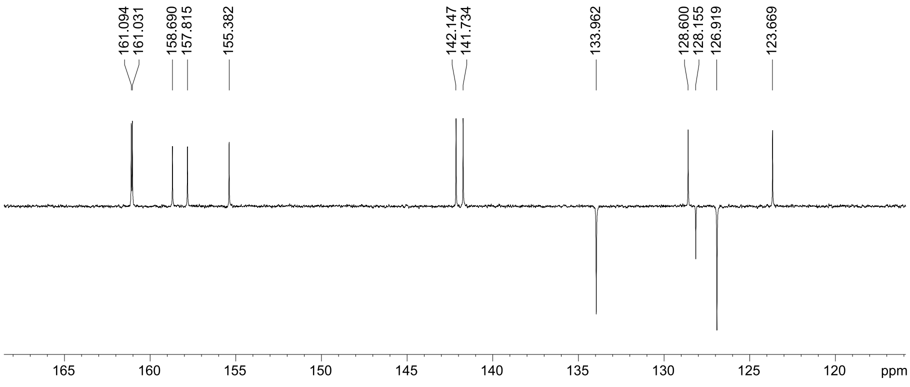

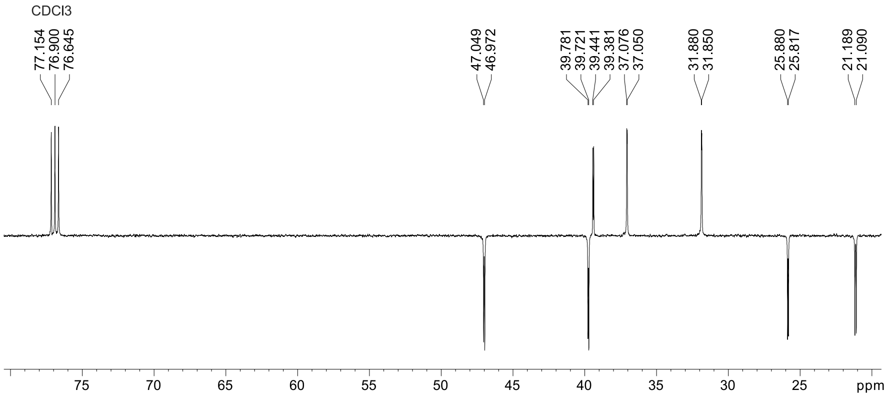

Current Data Parameters
NAME comp_8_lts-bs-1430-1
EXPNO 137
PROCNO 1

F2 - Acquisition Parameters
Date_ 20181015
Time 13.49
INSTRUM spect
PROBHD 5 mm Multinucl
PULPROG jmod
TD 65536
SOLVENT CDCl3
NS 516
DS 4
SWH 30303.031 Hz
FIDRES 0.462388 Hz
AQ 1.0813940 sec
RG 3251
DW 16.500 usec
DE 6.00 usec
TE 300.0 K
CNST2 145.0000000
CNST11 1.0000000
D1 4.00000000 sec
d20 0.00689655 sec
DELTA 0.00001184 sec
TD0 11111

======== CHANNEL f1 ========
NUC1 13C
P1 9.30 usec
p2 18.60 usec
PL1 5.00 dB
SFO1 125.7720754 MHz

======== CHANNEL f2 ========
CPDPRG2 waltz16
NUC2 1H
PCPD2 76.50 usec
PL2 -3.00 dB
PL12 13.00 dB
SFO2 500.1320005 MHz

F2 - Processing parameters
SI 131072
SF 125.7578122 MHz
WDW EM
SSB 0
LB 2.00 Hz
GB 0
PC 1.00

(1'*R*,3'*R*,8'*R*,10'*R*)-2',2',9',9'-tetramethyl-1',2',3',4',7',8',9',10'-octahydro-1*H*,3*H*-spiro[perimidine-2,12'-[1,3:8,10]dimethanocyclopenta[2,1-b:3,4-b']diquinoline] (9)

^{1}H NMR spectrum:

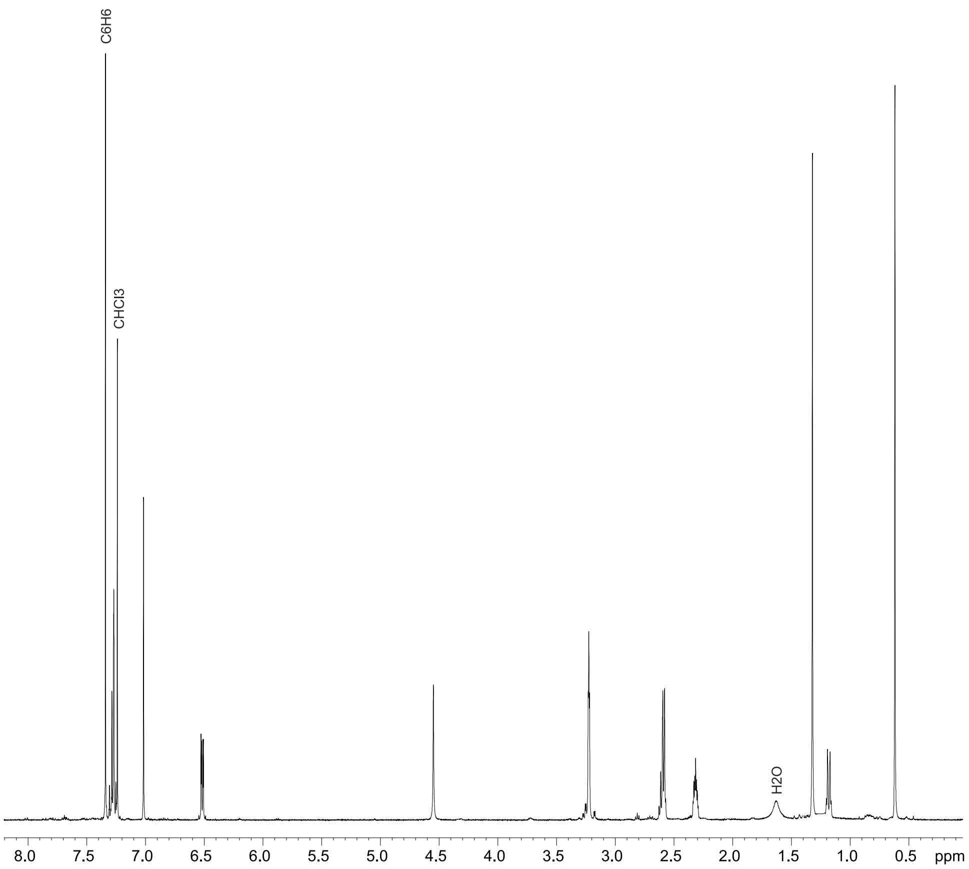

Current Data Parameters
NAME comp_9_l t s- bs- 1505-
EXPNO 1
PROCNO 1

F2 - Acquisition Parameters
Date_ 20190417
Time 17.51
INSTRUM spect
PROBHD 5 mm QNP 1H/13
PULPROG zg30
TD 32000
SOLVENT CDCl3
NS 16
DS 2
SWH 8802.817 Hz
FIDRES 0.275088 Hz
AQ 1.8176500 sec
RG 203
DW 56.800 usec
DE 6.50 usec
TE 293.4 K
D1 5.00000000 sec
TD0 11111

======== CHANNEL f1 ========
NUC1 1H
P1 13.70 usec
PL1 0 dB
PL1W 9.52005005 W
SFO1 400.1340013 MHz

F2 - Processing parameters
SI 65536
SF 400.1300184 MHz
WDW GM
SSB 0
LB -0.50 Hz
GB 0.3
PC 1.40

^{13}C NMR spectrum:

J-modulation,
broad-band decoupling

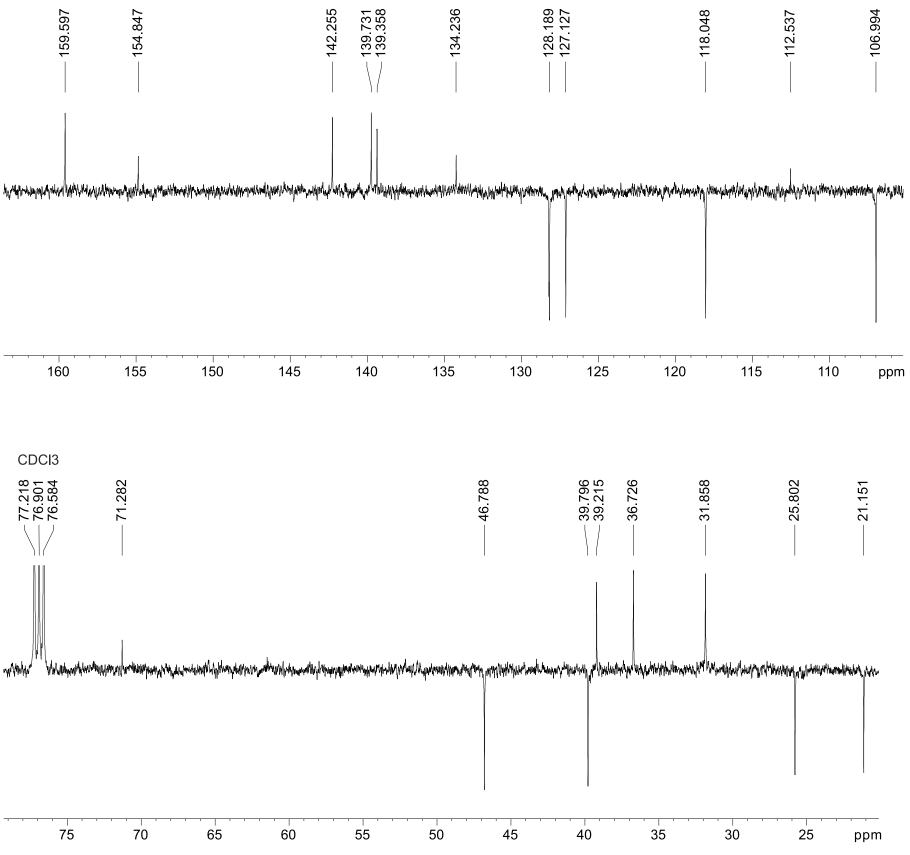

Current Data Parameters
NAME comp_9_lts-bs-1505-8
EXPNO 137
PROCNO 1

F2 - Acquisition Parameters
Date_ 20190418
Time 8.11
INSTRUM spect
PROBHD 5 mm QNP 1H/13
PULPROG jmod
TD 65536
SOLVENT CDCl3
NS 1232
DS 2
SWH 24038.461 Hz
FIDRES 0.366798 Hz
AQ 1.3631988 sec
RG 203
DW 20.800 usec
DE 6.50 usec
TE 293.3 K
CNST2 145.0000000
CNST11 1.0000000
D1 5.00000000 sec
D20 0.00689655 sec
TD0 11111

======== CHANNEL f1 ========
NUC1 13C
P1 9.60 usec
P2 19.20 usec
PL1 0 dB
PL1W 36.42235184 W
SFO1 100.6243395 MHz

======== CHANNEL f2 ========
CPDPRG2 waltz16
NUC2 1H
PCPD2 86.00 usec
PL2 0 dB
PL12 16.00 dB
PL2W 9.52005005 W
PL12W 0.23913284 W
SFO2 400.1316005 MHz

F2 - Processing parameters
SI 131072
SF 100.6127826 MHz
WDW EM
SSB 0
LB 2.00 Hz
GB 0
PC 1.40

6-chloro-N1-((1*R*,3*R*,8*R*,10*R*)-2,2,9,9-tetramethyl-3,4,7,8,9,10-hexahydro-1*H*-1,3:8,10-dimethanocyclopenta[1,2-*b*:5,4-*b*']diquinolin-12(2*H*)-ylidene)naphthalene-1,5-diamine (11)

^{1}H NMR spectrum:

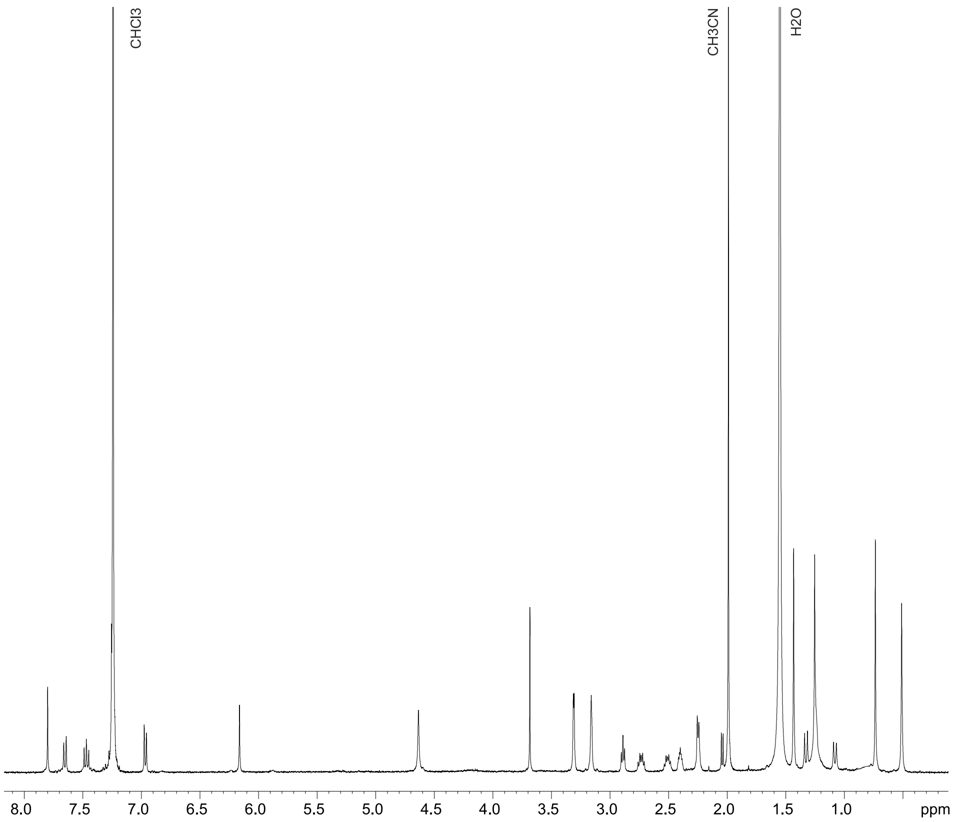

Current Data Parameters
NAME comp_11_l t s- esv0407
EXPNO 1
PROCNO 1

F2 - Acquisition Parameters
Date_ 20190628
Time 15.31
INSTRUM spect
PROBHD 5 mm QNP 1H/13
PULPROG zg30
TD 32000
SOLVENT CDCl3
NS 200
DS 2
SWH 8802.817 Hz
FIDRES 0.275088 Hz
AQ 1.8176500 sec
RG 203
DW 56.800 usec
DE 6.50 usec
TE 294.3 K
D1 5.00000000 sec
TD0 11111

======== CHANNEL f1 ========
NUC1 1H
P1 13.70 usec
PL1 0 dB
PL1W 9.52005005 W
SFO1 400.1340013 MHz

F2 - Processing parameters
SI 131072
SF 400.1300179 MHz
WDW EM
SSB 0
LB 0.30 Hz
GB 0
PC 1.00
